# A Narrative Review of Right Heart Dysfunction in Pulmonary Embolism: Current Insights and Clinical Perspectives

**DOI:** 10.7759/cureus.105115

**Published:** 2026-03-12

**Authors:** Rida Ilyas, Sana Masood, Ambreen Fatima

**Affiliations:** 1 Gastroenterology, South Tees NHS Foundation Trust-James Cook University Hospital, Middlesbrough, GBR; 2 General Internal Medicine, South Tees NHS Foundation Trust-James Cook University Hospital, Middlesbrough, GBR; 3 Community Medicine, Shalamar Medical and Dental College, Lahore, PAK

**Keywords:** anti-coagulation, ctpa, deep venous thrombosis, echocardiography, pulmonary embolism, right heart strain, risk stratification, thromboembolism

## Abstract

Pulmonary embolism (PE) often presents with non-specific symptoms that make the diagnosis challenging, but the identification of right heart strain (RHS) can help determine the disease severity. Therefore, timely identification and diagnosis of RHS in patients with pulmonary embolisms is critical for accurate risk categorization and appropriate management. The review mainly concentrates on acute pulmonary embolism and right‑heart strain assessment, risk stratification, and management using European Society of Cardiology (ESC) 2019 guidelines developed in collaboration with the European Respiratory Society (ERS). Right‑heart strain is diagnosed by imaging signs such as right‑ventricular dilation, interventricular septal flattening, McConnell’s sign, TAPSE < 16 mm, or an RV/LV ratio > 1 on CTPA, together with elevated biomarkers (NT‑proBNP, troponin I, and MPV). These biochemical markers are chosen because they rise with right‑ventricular overload or injury.

The aim of this study is to identify a literature gap in the diagnosis of RHS in the context of PE. Various biochemical markers and imaging modalities to diagnose RHS with PE are assessed. Despite the available tools, the current diagnostic approaches remain limited due to the absence of standardized measurement techniques and guidelines for right heart strain assessment on CTPA and echocardiography. Standardization of the assessment of right heart structure and function should be established, as this significantly impacts patient management. This study invites the researchers and international collaboration to set guidelines for the diagnosis of right heart strain in the context of pulmonary embolism to improve patient management and outcomes. This will also help to reduce mortality worldwide.

A narrative review of existing studies, imaging techniques, and blood test markers was conducted to highlight existing gaps and call for future guidelines. A literature search was conducted using the Google Scholar database for articles published from 2000 to the most recent ones. Keywords included 'pulmonary embolism,' 'right ventricular dysfunction,' 'right heart strain,' 'thromboembolism,' 'deep venous thrombosis,' 'CT pulmonary angiogram,' 'echocardiography,' and 'anticoagulation.' No limitations were placed on the search to maximize inclusion of relevant literature. Duplicates and non-English articles were excluded.

## Introduction and background

Pulmonary embolism (PE) is described as a blockage of one or more branches of the pulmonary arterial system by a thrombus. This thrombus typically originates from deep venous thrombosis (DVT) of the lower limbs. If pulmonary embolism is not treated in time, it may lead to right ventricular disorders due to chronic thromboembolic pulmonary hypertension (CTEPH) [1]. CTEPH is defined as a chronic complication of PE due to persistent pulmonary hypertension secondary to obstruction, even after six months of anticoagulation [2]. Right ventricular (RV) dysfunction refers to impaired systolic performance of the right ventricle. This impairment causes reduced right ventricular filling and diminished ventricular flow output [3]. It leads to increased pulmonary vascular resistance and eventually, right ventricular failure. This advanced stage is characterized by hemodynamic instability and reduced cardiac output [4]. Right heart strain (RHS) refers to acute imaging or biochemical evidence of RV pressure overload in the setting of PE. 

Acute pulmonary embolism (PE) is a major cause of morbidity and mortality in the United States and Europe, accounting for 100,000 and 300,000 deaths annually, respectively. Massive PE, characterized by systemic hypotension or cardiogenic shock, is associated with an in-hospital mortality of 25-50%. Whereas submassive PE, characterized by RV dysfunction without hypotension, has an in-hospital mortality rate of 3-15% [5]. PE and DVT survivors face a higher chance of death, which can persist for as long as 30 years after diagnosis. Also, the cumulative incidence of CTEPH has previously been estimated to be 2.2% 10 years after VTE [6]. Patients can present with various clinical manifestations, including syncope in 5-13%. First reported in the 1970s, syncope was associated with evidence of right ventricular (RV) strain on electrocardiograms [7]. According to Heidinger et al., right ventricular (RV) systolic dysfunction is associated with increased short-term mortality in patients with PE [8].

An important risk factor for PE is deep venous thrombosis (DVT). Pulmonary emboli originate when clots are dislodged from the deep veins, most commonly from the calf veins, followed by the femoropopliteal, and very rarely from the iliac veins, and block the vasculature of the pulmonary arteries, causing an increase in the pulmonary artery pressure and increasing stress on the right side of the heart. PE can also originate from the upper limb, although this occurs much less frequently than from the lower limb. Lower limb venous thromboembolism has a high incidence (15-32%) of causing PE, whereas the incidence of upper extremity causing PE is only 6% [9]. The first 30 days after diagnosis of pulmonary embolism are critical, as the risk of death in such patients is 31% [10].

Right heart strain in PE causes a reduction in cardiac output, reduced contractility, and the possibility of the formation of chronic thromboembolic pulmonary hypertension [4]. An increase in pulmonary vascular resistance due to a large thrombus leads to an increase in right ventricular afterload, but right ventricular walls are thin and cannot bear a sudden increase in the afterload [11]. As the stress on the heart muscle increases, particularly on the right side, it leads to hemodynamic instability, which is biochemically reflected by markers of NT-proBNP, troponin I, and MPV. It is worth noting that right ventricular strain and right heart thrombus often coexist. Free-floating right heart thrombi are rare but a major contributing factor to pulmonary embolism. These emboli eventually become responsible for hemodynamic abnormalities when they block pulmonary artery branches [12]. To minimize the mortality rate in pulmonary embolism, early detection and treatment are essential [13]. Moreover, right ventricular strain is critical to detecting the severity of pulmonary embolism [14]. Arterial blood gas (ABG), brain natriuretic peptide (BNP), and troponin are lab markers used to diagnose or rule out heart strain in patients with pulmonary embolism. Whereas computed tomography pulmonary angiography is the gold standard for detecting embolism. While transthoracic echocardiography (TTE) is one of the main imaging modalities that are specific to detecting right heart strain, it can also critically assess the pressure overload in the right ventricle [15].

Approach to right heart strain and pulmonary embolism

Assessment and management of multiple risk factors associated with right heart strain and pulmonary embolism are crucial. Early risk stratification is mandatory to rule out the disease’s root cause and to minimize the mortality and morbidity rate [15]. Right heart strain is significantly associated with pulmonary embolism as a compensatory mechanism [16].

Clinical evaluation

Although the clinical presentation of pulmonary embolism with symptoms of dyspnea, cough, chest discomfort, and hemoptysis can be very nonspecific and vague, the presentation with syncope, hemodynamic instability, and circulatory compromise is strongly suggestive of right heart strain. This is an infrequent yet significant clinical finding causing an inability to maintain stable blood flow. In addition to common signs of tachycardia and tachypnea in PE, the presence of jugular venous distension and the loud P2 (pulmonic) component of the second heart sound strongly indicate pulmonary hypertension and right heart strain. However, the presence or absence of these signs is not specific to including/excluding PE and RHS [17].

Laboratory markers

Right heart strain in pulmonary embolism is a potentially life-threatening condition that needs to be diagnosed early to minimize the risk of mortality. Studies show that out of eight lab markers that can be beneficial to identify PE, three of them are of significance and widely used for screening. These markers are mean platelet volume (MPV), NT-proBNP, and troponin I [18].

A raised MPV is significantly observed in early detection of PE as it indicates the presence of thrombosis in venous thromboembolism. The ratio of MPV to lymphocyte count is an important lab marker for the prediction of prognosis and mortality in PE. Since a full blood count is routinely requested in patients with PE, this biomarker is cost-effective and easy to calculate. However, Yurtseven and Ensarioğlu (2024) suggested that MPV could not be reliably used for risk assessment of PE patients [19]. Despite the variation in the literature regarding the estimation of MPV’s role in PE, MPV shows a strong association with right ventricular dysfunction and myocardial injury in acute PE. According to an observational study, MPV >10.9 fl indicated significantly increased mortality, especially during the first seven days after admission [20].

N-terminal-pro-brain natriuretic peptide (NT-proBNP) is a crucial marker for right ventricular dysfunction in patients suffering from pulmonary embolism. This serum analysis provides a fundamental piece of information for patients undergoing surgical treatment for PE. This cost-effective tool helps in risk stratification for PE, as increased levels of NT-proBNP indicate increased risk of complications pre- and postoperatively [21]. Chen et al. found that age-adjusted cutoff values for NT-proBNP in the context of right ventricular dysfunction in PE are 356 pg/ml, 526 pg/ml, and 647 pg/ml in ages <55, 55-69, and >70 years, respectively [22]. 

Troponin I is a regulatory protein of cardiac muscles. It gets released into the bloodstream if there is any destruction of cardiac myocytes. In PE with RHS, troponin I is considered an important biomarker, as it helps in risk stratification. An increased level of troponin I indicates massive RV dysfunction secondary to overload, ischemia, or necrosis caused by pulmonary artery obstruction [23]. Keller et al. report that cutoff values for troponin I for right ventricular dysfunction and submassive PE are 0.01 ng/mL with a negative predictive value of 73% [24]. 

Imaging

Right heart strain is specifically diagnosed using tools such as a transthoracic echocardiogram (TTE) and computed tomography (CT). On an echocardiogram, right heart strain appears as dilation of the right ventricle and interventricular septal flattening. Moreover, Zhang et al. (2024) [25] mentioned in their study that McConnell’s sign is the echocardiographic finding that indicates right ventricular free wall hypokinesia with a hyperdynamic right ventricular apex, and tricuspid annular plane systolic excursion (TAPSE) is less than 16 mm on the transthoracic echocardiogram [26]. This is because TAPSE <16 mm represents reduced right ventricular systolic function, and it has clinical relevance as a sign of severe RV strain and increased risk of hemodynamic deterioration in pulmonary embolism [27].

Cardiovascular magnetic resonance (CMR) is considered the gold standard to evaluate the right ventricular anatomical and physical abnormalities; however, it is expensive and more time-consuming. It is advised to be used when the echocardiographic results give a vague diagnostic picture [28].

In order to find the underlying cause of PE, an ultrasound of the lower limbs was previously preferred [29]. Currently, CTPA remains the gold standard for pulmonary embolism diagnosis, and it can also give hints about right heart strain [30]. CTPA has the capability to visualize any intravascular thrombi as well as evaluate signs of RHS by measuring the RV/LV ratio. Additionally, CTPA-derived markers of RHS remain important in assessing PE severity [31]. The RV/LV ratio is a quantitative measure, and a ratio above 1 signifies right ventricular dysfunction [32].

Risk stratification

Patients with right heart strain in pulmonary embolism face a high risk of clinical deterioration and death, which is why identifying the individual risk factors is important for providing timely and appropriate treatment to the patients. In order to assess the need for hospitalization and treatment planning, patients are divided into three groups based on severity: high-risk, intermediate-risk, and low-risk [33]. Several indices are devised to assess severity, such as the pulmonary embolism severity index (PESI), simplified pulmonary embolism severity index (sPESI), Bova, and FAST score. These scores evaluate high-risk patients; however, the low-risk patients are assessed using the Hestia score [34].

Previously, an index named PESI was used, in which patients were investigated on 11 clinical features. However, it was then modified to sPESI using only six significant clinical features to assess the severity of PE. These features included age over 80, previous cancer history, systolic blood pressure under 100 mmHg, chronic cardiopulmonary disease, oxygen saturation less than 90%, and heart rate over 110 beats per minute. This score evaluated the 30-day mortality risk. Next in line, the Hestia score was developed for low-risk patients who could be treated safely outside the hospital, allowing for outpatient treatment at home [35].

Particularly, in patients with RHS and hemodynamic instability, the Bova score is considered a more effective assessment tool because it includes RHS as one of its components, and it also measures the right heart dysfunctions and other cardiac parameters. This prognostic tool predicts early mortality and helps in devising a perfect treatment plan [36]. A Bova score of >4 is linked with higher in-hospital and one-year mortality in pulmonary embolism patients [37]. The FAST score is also considered novel as it gives deep insight into the heart fatty acid-binding protein, syncope, and tachycardia. However, FAST is only used as a prognostic score for short-term outcomes in PE [38].

Table 1 highlights a comparison of key aspects of the Bova and FAST scores for assessment of severity and prognosis in patients with PE and RHS. These tools evaluate clinical parameters, comorbidities, cardiac biomarkers, and right ventricular dysfunction to identify patients at higher risk of complications. Their application aligns with the 2019 European Society of Cardiology (ESC) guidelines, which classify patients into high, intermediate-high, intermediate-low, and low-risk groups based on hemodynamic status, biomarkers, and right heart dysfunction, thereby guiding decisions regarding hospitalization and treatment [39].

**Table 1:** Comparison of Bova and FAST Scores for Risk Stratification in Pulmonary Embolism with Right Heart Strain [40,41] 40. Bova C, Sanchez O, Prandoni P, Lankeit M, Konstantinides S, Vanni S, Jiménez D. Identification of intermediate-risk patients with acute symptomatic pulmonary embolism. Eur Respir J. 2014 Sep;44(3):694–703. doi:10.1183/09031936.00006114. 41. Hobohm L, Becattini C, Konstantinides SV, et al. Validation of a fast prognostic score for risk stratification of normotensive patients with acute pulmonary embolism. Clin Res Cardiol. 2020;109:1008–17. doi:10.1007/s00392-019-01593-w.

Feature	Bova score	FAST/ Modified FAST score
Purpose	Helps to predict 30 days’ mortality in normotensive patients with acute PE.	Predicts deterioration and adverse in-hospital outcomes: - PE-related death - mechanical ventilation - cardiopulmonary resuscitation - administration of catecholamines.
Variables included	Systolic BP, Elevated Cardiac troponin, RV dysfunction (TTE/CT), Heart rate	Heart rate, Syncope, Elevated troponin
Scoring criteria	0-2 points: Stage 1, low risk; 3-4 points: Stage 2, intermediate risk; >4 points: stage 3, high risk.	<3 points: low risk for adverse in-hospital outcome; >3 points: Intermediate- high risk for adverse in-hospital outcome.
Benefits	Simple to use, integrates imaging (TTE/ CT) and biochemical markers to assess right ventricular dysfunction, facilitates Staging of PE- low risk, intermediate risk and high risk, Predicts PE-related mortality at 30 days, Includes RV dysfunction in risk stratification, Well established in literature.	Can be used for haemodynamically unstable patients with PE, Useful tool to predict inpatient deterioration and need for establishing escalation plans.
Limitations	Hemodynamically unstable patients are not eligible for Bova scoring. It can only be used in normotensive patients with confirmed acute PE.	Less validated, variable definitions across studies which makes generalisation challenging. Inclusion of only biochemical markers for RV dysfunction. It may under-represent the risk of RHS in patients.

Management of pulmonary embolism with right heart strain

The management of patients with pulmonary embolism is critical and challenging. Acute management of PE is done by careful evaluation and stabilization of the patient at the time of clinical presentation. For high-risk patients, such as PE with hemodynamic instability with RHS, the available treatment modalities can be catheter-directed thrombolysis, percutaneous thrombectomy, mechanical circulatory support, and extracorporeal membrane oxygenation [42]. A common first-line treatment to avoid the propagation and migration of blood clots in the body is the initiation of anticoagulant therapy, including unfractionated heparin or low molecular weight heparin. However, as per the severity of the disease, thrombolysis can be commenced, through which fibrinolytic agents are administered to dissolve the clots rapidly and effectively. However, thrombolytic therapy is contraindicated in patients with active bleeding, recent surgery, or a history of hemorrhagic stroke [43]. Moreover, for long-term management, direct oral anticoagulants (DOACs), e.g., rivaroxaban, apixaban, etc., are used. The use of DOACs is preferred over the use of warfarin because warfarin needs continuous and careful monitoring as compared to rivaroxaban and apixaban [44]. Surgical or catheter direct thrombolysis is preferred only when thrombolytic therapy does not respond or is contraindicated. The purpose is to restore cardiac and pulmonary blood flow [45]. In certain patients who have contraindications to anticoagulation or recurrent embolism even after therapy, an inferior vena cava (IVC) filter may be considered to prevent further migration of thrombi to the lungs [46]. While treating the patient for PE, it is significant and obvious to consider right ventricular dysfunction, and imperative measures are taken to relieve the right heart strain [4].

## Review

Discussion

Pulmonary embolism is, and has always been, a very commonly encountered emergency in hospitals. It carries a high disease burden in terms of high rates of mortality and morbidity. It carries a significant disease burden and is accounted for as the third leading cause of death after myocardial infarction and stroke [47].

It is estimated from European data that around 45% of patients with DVT develop PE [48], and around 3.8% of patients will have pulmonary hypertension (chronic thromboembolic pulmonary hypertension) and a gradually progressive right heart failure [49].

NT pro-BNP is also used to evaluate the strain on the heart due to myocardial stretch in acute PE. Plasma levels of NT-pro-BNP reflect the severity of RV dysfunction. Studies report that patients with elevated NT-pro BNP with acute PE had a 10% risk of early death and a 23% risk of adverse clinical outcome [50].

Apart from NT pro-BNP as a blood test, MPV and troponin I are also very significant prognostic markers used to assess the burden of PE on the cardiac muscle. In a study, it has been found that around 70% of patients with PE had raised troponin I and that this is associated with right heart dysfunction. A surge in troponin I within 10 hours has been associated with adverse outcomes and high risk of early mortality as compared to individuals with near-normal troponin I levels. Hence, early measurement of troponin I may improve the overall outcome of PE by enabling early risk stratification [51].

MPV levels have been seen to rise in acute PE, and raised levels reflect the stress of the heart. This is because larger platelets are more active and contain more prothrombotic factors, which allow them to release chemicals that help platelets aggregate and form clots more quickly. Therefore, a higher mean platelet volume (MPV) suggests an increased tendency for thrombosis and may potentially indicate worse outcomes. From a clinical point of view, establishing a clear MPV cut-off could help identify disease severity, thrombotic risk, mortality risk, and treatment response [52].

It has been a commonly encountered debate for the ideal choice of diagnostic modality when it comes to establishing the effect of PE on the heart. While CTPA provides reliable objective measurements such as RV:LV ratio, echocardiography allows bedside functional assessment but is operator-dependent, and CMR offers the most precise anatomical and functional evaluation of right heart strain but is less practical acutely due to cost and availability [11]. CTPA vs. ECHO stands as the investigation of choice to determine the severity of right heart strain. In a study, CTPA has been used to predict adverse outcomes based on mild, moderate, or severe PE (in relation to its effect on the right heart) [53].

Echocardiography is a useful tool in assessing cardiac function at the bedside, which is seen as hypokinesia of the right ventricular wall, predominantly to ascertain RHS. Echocardiography is operator-dependent, so it lacks reliability.

In terms of management, the mainstay of treatment for PE is anticoagulation. Depending on the severity of PE, i.e., the effect on the heart, treatment should be warranted to address both. Outpatient PE treatment is comprised of either LMWH, DOACs, or warfarin, depending on further underlying risk factors leading to PE. In the instance of hemodynamic compromise and suspicion of PE, a thrombolytic agent such as alteplase is used. The duration of treatment also holds paramount importance, depending on whether it is provoked or unprovoked PE and further underlying risk factors.

Limitations of current evidence

This is a narrative review and does not hold any formal quantitative statistics. Variations in imaging techniques and biomarkers limit direct comparison across studies. Furthermore, most of the available literature is from observational cohorts rather than randomized controlled trials.

## Conclusions

A pulmonary embolism is the third most common cause of death owing to its effect on the major organs of the body and impeding the blood flow in an acute scenario. Its effects on the heart can be lasting due to stress on the right side of the heart. There have been no standardized cutoffs and measurement techniques in CTPA and echocardiogram for RHS, which limits the incorporation of RHS in clinical practice. This lack of standardized RHS assessment makes the linkage between right heart strain and mortality outcomes complicated. Studies have shown that patients with imaging evidence of RHS have significantly higher short-term mortality and adverse clinical outcomes compared to those without RHS. Hence, a standardization call to the international collaboration has been issued by the expert consensus for the standardization of the assessment of right heart structure and function. Bova and FAST scoring include the parameter of right heart dysfunction in the scoring system, and its use in clinical practice would likely make an impact. Integrating Bova and FAST scores in clinical decisions could refine the treatment strategies and also improve prognosis prediction. Right heart strain in PE is not for diagnosis but holds an integral place in risk stratification and future implications for the patient. Despite its crucial role, there has been a difference in opinion, and no standardized guidelines define it. In the future, the research should warrant a standardized approach in diagnosing RHS in PE and the ceiling of care based on it. Future standardization would be able to use the imaging parameters or biomarkers, with research priorities, for guideline development and multicenter validation.
